# Natural Pyrrhotite as a Catalyst in Prebiotic Chemical Evolution

**DOI:** 10.3390/life3030502

**Published:** 2013-08-28

**Authors:** Alejandra López Ibáñez de Aldecoa, Francisco Velasco Roldán, César Menor-Salván

**Affiliations:** 1 Centro de Astrobiología (CSIC-INTA), 28850 Torrejón de Ardoz, Madrid 28850, Spain; E-Mail: lopezima@cab.inta-csic.es; 2 Departamento de Mineralogía y Petrología, Facultad de Ciencia y Tecnología, Universidad del País Vasco, Bilbao 48080, Spain; E-Mail: francisco.velasco@ehu.es

**Keywords:** pyrrhotite, life origin, pyruvate, lactate, reductive carboxylation, thioesters

## Abstract

The idea of an autotrophic organism as the first living being on Earth leads to the hypothesis of a protometabolic, complex chemical system. In one of the main hypotheses, the first metabolic systems emerged from the interaction between sulfide minerals and/or soluble iron-sulfide complexes and fluids rich in inorganic precursors, which are reduced and derived from crustal or mantle activity. Within this context, the possible catalytic role of pyrrhotite, one of the most abundant sulfide minerals, in biomimetic redox and carbon fixation reactions was studied. Our results showed that pyrrhotite, under simulated hydrothermal conditions, could catalyze the pyruvate synthesis from lactate and that a dynamic system formed by coupling iron metal and iron-sulfur species in an electrochemical cell could promote carbon fixation from thioacetate esters.

## 1. Introduction

Since the relationship between hydrothermal systems and the origins of life was first proposed [1,2] the complete geochemical perspective of this relationship has been described in detail [3]. The experiments that have replicated sulfide rich hydrothermal solution chemistry have confirmed the potential of these systems to synthesize organic molecules through carbon fixation. Ferrous sulfide minerals, which usually precipitate in the submarine hydrothermal systems, that are candidate environments for the origin of life, play a key role in this process. These iron minerals include pyrrhotite, as well as mackinawite, greigite, and violarite [4], which are unstable minerals under aerobic conditions. The hypothesis of a chemoautotrophic origin of metabolism, proposed by G. Wächtershäuser, suggested that iron sulfide surfaces could support an autocatalytic chemolithotrophic metabolism driven by the exergonic formation of pyrite (FeS_2_) from more reduced minerals, such as pyrrhotite or pentlandite, in the presence of free sulfide [5]. The result that synthetic (Fe,Ni)S catalyzed the synthesis of methyl mercaptan and acetic acid, using CO as an inorganic carbon source, reinforced the idea of a prebiotic chemistry associated with iron sulfide minerals, although the mineral phases involved in this experiment have not been identified [6]. In a parallel elaboration of the iron sulfide scenario, the M. Russell model [7] incorporated the idea that compartmentalization (with membranous FeS precipitates as ancestral compartments) is essential for the reactive concentration and the origin of a gradient dissipation based carbon fixation metabolism, which is driven by chemiosmotic and proton-motive forces [8,9]. The carbon fixation of organic material is an obvious prerequisite for life, and Earth’s current biochemistry maintains five autotrophic pathways in a metabolic strategy that is highly conservative in anaerobic autotrophic prokaryotes [10]. The characteristics and preservation of carbon fixation pathways in the extant biochemistry caused us to look back to the beginning of biological evolution associated with the development of an autocatalytic autotrophic biochemical system, in which transition metal complexes containing proteins play an essential role [11]. In particular, the role of iron sulfur proteins, which contain Fe-S clusters as active centers in the electron transfer reactions in the CO_2_ fixation pathways, and their occurrence in what are possibly the most primitive steps of oxidation of organic substrates, such as succinate, in the cell energy transduction machinery, have been the key evidence used to connect the origin of biochemistry and the geochemistry of the origin of life [12]. Thus, considering the structural similarity between the biological iron-sulfur clusters and the crystal structure of iron sulfide minerals [13], the biomimetic activity of synthetic soluble Fe-S clusters [14] and the highly preserved and ancient biochemical reactions involved could explain why Fe-S clusters are found in all biological systems and why iron sulfide clusters were chosen by nature rather than other metal clusters or organometallic catalysts. In this sense, Fe-S clusters could be “living fossils” that trace the origin of life to the mineral roots of biochemistry.

Using the idea of the construction of a model of protometabolism catalyzed by ancient iron-sulfur active centers, we focused on the origination of pyruvate metabolism on pyrrhotite matrix. We selected pyrrhotite, due to its stability relative to more reduced iron sulfide minerals and due to the central role that this mineral plays in the iron-sulfur geochemistry: its transformation in pyrite could provide redox energy to the system, could be a source of soluble FeS clusters [15], and its surface could have efficient catalyst and electron transfer properties.

Recently, it has been demonstrated that the interconversion of hydroxyl acids and keto acids could be catalyzed by the FeS/FeS_2_ system [16]. Using this reference, we tested the feasibility of using natural iron sulfide pyrrhotite to mimic oxidoreductase activity in the interconversion of pyruvic and lactic acids. The pyruvate is a central metabolite in the Archaea, Bacteria, and Eukarya kingdoms, and iron-sulfur enzymes are involved in the reactions that link pyruvate with carbon fixation pathways and with thioester biochemistry. In many anaerobic autotrophic organisms, the reductive carboxylation of acetyl-CoA to pyruvate could be catalyzed by pyruvate-ferredoxin oxidoreductase (PFOR), which acts as pyruvate synthase using the iron-sulfur protein ferredoxin as an electron donor [17]. This reaction connects the Wood-Ljundhal pathway with the reductive Krebs cycle to generate biosynthetic intermediates in the anabolic metabolism of Archaea, such as *Methanobacterium thermoautotrophicum*. The PFOR is an ancient heterotetrameric molecule present in all Archaea, autotrophic bacteria and anaerobic protozoa. The ancestral subunit is ferredoxin-like and binds iron-sulfur clusters [18], reinforcing the idea of its prebiotic origin. 

However, the reactivity of thioesters, their biochemical role, and their plausible prebiotic formation suggest that primitive analogs of coenzyme A thioesters could have been involved in the origin of biochemistry [19]. Similarly, it has been proposed that the reductive carboxylation of thioacetic acid using FeS as an electron donor to form pyruvic acid is a possible starting point for a prebiotic thio-analog of the reductive Krebs cycle [20]. The standard reduction potential of pyruvate synthesis by reductive carboxylation is Eº = −500 mV [10]. To drive the reductive carboxylation forward, low potential electron donors are needed. In this work, we explore the possibility of connecting the biochemical reductive carboxylation of thioesters to the geochemistry of the iron-sulfide system. Considering iron sulfide clusters and minerals as prebiotic analogs of ferredoxin iron-sulfur clusters and the iron metal/pyrrhotite/pyrite system as a source of reducing power, we performed the reductive carboxylation of ethyl thioacetate to model the more complex acetyl-CoA thioester. This constitutes a test for the pyrite-pulled intermediary metabolism proposed by Wächtershäuser and the suggested synthesis of pyruvic acid from thioacetic acid and CO_2_ [5,20,21]. 

## 2. Experimental Section

### 2.1. Pyrrhotite Mineral Characterization

**Figure 1:**
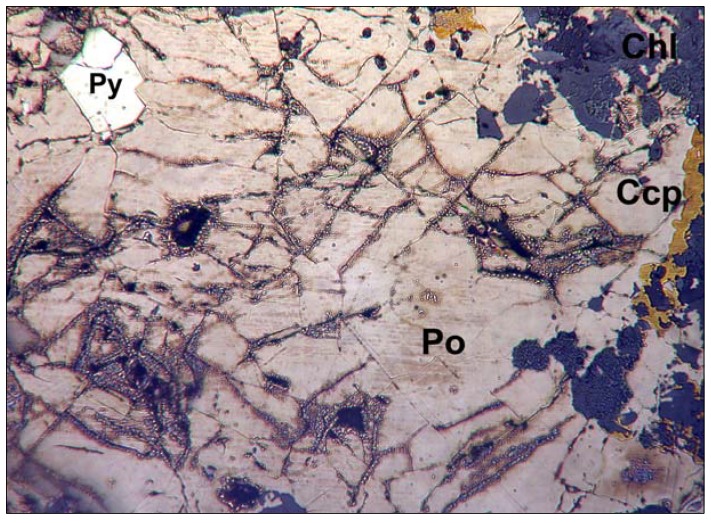
Texture of the pyrrhotite ore assemblage from the Gualba deposit used in the experiments after separation of the accompanying minerals. Po: pyrrhotite; Ccp: chalcopyrite; Py: pyrite; Chl: chlorite minerals. Note the appearance of products of alteration after polishing close to the microfractures.

All of the pyrrhotite samples used were from the Gualba deposit (Barcelona, Spain), a Fe-Cu polymetallic skarn formed by the replacement of the Cambrian-Ordovician carbonate rocks after intrusion of a body of granodioritic composition of Hercynian age [22]. The pyrrhotite was characterized by electron and optical microscopy, electron microprobe analysis (EMPA-WDS), and X-ray diffraction (XRD). The composition was determined to be Fe_0.89_S, and the mineral consists of a mixture of hexagonal and monoclinic polytypes. The ore was selected for its massive character and purity, which minimized the trace mineral and organic carbon contents. The Gualba pyrrhotite is strongly ferrimagnetic; this characteristic has been used for the isolation of pyrrhotite from the ore paragenesis, which includes traces of pyrite, chalcopyrite, and very subordinate amounts of arsenides and non-metallic minerals of the mica and chlorite groups (Figure 1). The nickel content of the pyrrhotite from the Gualba quarries was 0.012–0.018 wt%, with negligible copper and cobalt.

**Figure 2:**
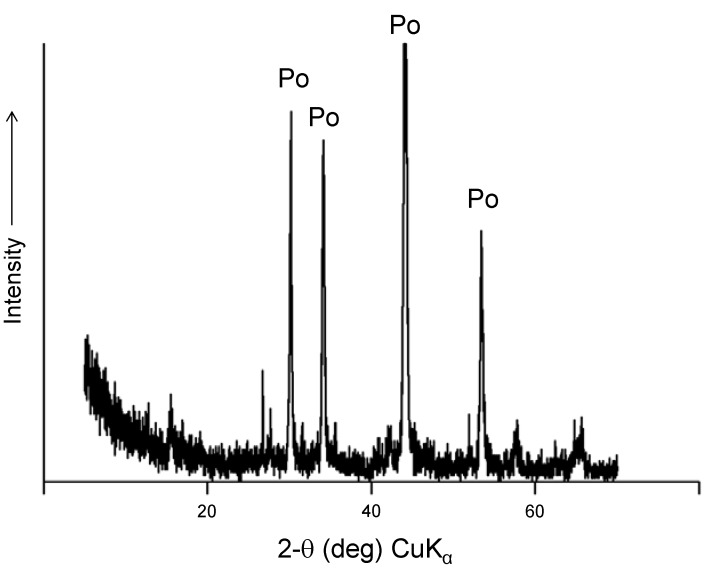
Powder X-ray diffraction of the Gualba pyrrhotite concentrate after magnetic separation and washing. The presence of pyrite and other minerals is negligible.

The ore samples were crushed and pyrrhotite was magnetically separated; the pyrrhotite powder was analyzed by XRD before the experiments to assure that it was pyrite free (Figure 2). To eliminate potential organic contaminants, the nearly pure pyrrhotite concentrate was extracted first with dichloromethane and then with methanol and then dried and stored in an anoxic atmosphere. 

### 2.2. Pyrrhotite in Lactate-Pyruvate Oxidoreduction

All of the experiments were conducted in sealed 22 mL headspace vials and in a glove box with a nitrogen atmosphere. Each vial was filled with 1 mmol of crushed pyrrhotite, 1 mmol of elemental sulfur (S), and 5 mM of buffer phosphate (with different pH: 2, 5 and 8). Subsequently, 100 µmol of pyruvic acid or 200 µmol of lactic acid were added, together with 0.5 mmol of sodium sulfide and 0.5 mmol of sulfuric acid, which served to provide the H_2_S. The reactions were performed at 130 °C for a period of 5 h. Finally, the aqueous phase was separated and analyzed by gas chromatography-mass spectrometry. The remaining mineral fraction was characterized by XRD to determine the mineral phase transformations. 

### 2.3. Metallic Iron/Pyrrhotite in Lactate-Pyruvate Synthesis

**Figure 3:**
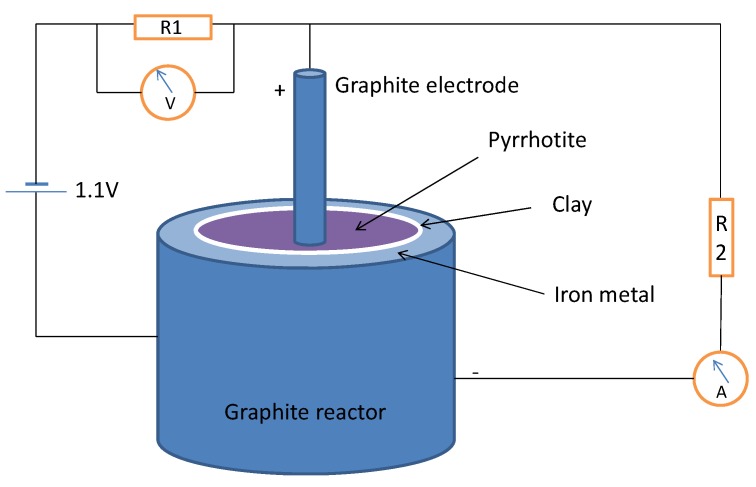
Electrochemical cell designed to perform the reductive carboxylation of thioacetate ester. See text for details and resistor values.

An electrochemical cell has been constructed using a cylindrical graphite reactor filled with granulated iron metal under a 10 mM sodium bisulfate solution (pH 5.5), a microporous clay barrier and pyrrhotite wet paste formed by pyrrhotite powder, a 50 mM ammonium bicarbonate solution containing 1 mmol of sodium sulfide (pH 9) and, optionally, 1 mmol of hydroquinone. Previously, 1 mmol of ethyl thioacetate was adsorbed by the pyrrhotite powder. A graphite electrode inserted in the pyrrhotite constitutes the cathode. The constructed cell showed a direct measured potential of 0.400 V to 0.835 V, depending on the composition and the pH of the solutions, using the reactor as the anode and the graphite electrode as the cathode. The cell was connected to the circuit depicted in Figure 3. The value of resistor R1 is 1.5 KΩ and the value of resistor R2 is 100 KΩ. The voltmeter measures the external voltage source plus the voltage provided by the iron/pyrrhotite cell. The system was connected under an anoxic (N_2_) atmosphere in a glove box; all of the instruments and materials used were first sterilized to minimize bacterial contamination. After 72 h, the reactor content was extracted with hot water, and the solution was filtered through a cation exchange resin column (Dowex 50w X8) in H^+^ form to remove dissolved iron and other metallic cations. The clear, slightly yellowish solution was freeze-dried and stored at −20 °C until the chromatographic analysis was performed.

### 2.4. Gas Chromatography-Mass Spectrometry

The solid, freeze-dried samples were derivatized with N,O-bis(trimethylsilyl) trifluoroacetamide (BSTFA) with 1% trimethylchlorosilane (TMCS, provided by Pierce, Rockford, IL, USA). Briefly, 1 mg of dried sample was combined with 0.1 ml of BSTFA+TMSC in a dry glass vial, stirred, and heated at 60 °C for 3 h. Following that, 1 µL of sample was transferred to an Agilent GC 6850A gas chromatograph in the splitless mode with the injection port at 290 °C. The analysis was performed using an HP-5MS (Agilent), 5% phenyl-95% methylsiloxane capillary column (30 m x 0.25 mm i.d., 0.25 µm film). Helium was used as the carrier gas at a flow rate of 1.1 ml/min. The oven temperature, initially at 40 °C for 1.5 min, was ramped up to 130 °C at a rate of 5 °C/min. Following this, the temperature was ramped up to 180 °C at 10 °C/min and held there for 10 min. Subsequently, the temperature was raised to 220 °C at a rate of 20 °C/min and held there for 15 min. In the final step, the temperature was raised to 300 °C at a rate of 10 °C/min and held there for 18 min. 

Mass spectrometric analysis was performed using an Agilent 5975C VL MSD quadrupole in EI+ mode with an ionization energy of 70 eV. The ion source temperature was set to 230 °C and the quadrupole was set to 290 °C. Identification of compounds was performed in scan mode with a range of 45–650 amu. 

The identified compounds were confirmed against authentic standard (provided by Sigma-Aldrich) mass spectra and retention times. Controls performed using mixtures of standards were used to identify possible derivatization artifacts and to avoid ambiguities. Those peaks with less than a 90% match probability in the database and/or tentatively or ambiguously identified were considered unidentified and are not discussed in this paper.

## 3. Results and Discussion

### 3.1. Oxidoreductase Activity of Pyrrhotite

In a prebiotic scenario, the synthesis of pyruvic acid is of great interest. Many studies have focused on the role of this keto acid in the synthesis of other organic compounds with metabolic relevance, such as acetic acid, methylsuccinic acid and other cyclized compounds [23], oxalacetic acid, acetoacetic acid, fumaric acid, and succinic acid [24]. In the first steps toward the formation of the protometabolic system prior to the origin of living biochemistry, the redox reactions catalyzed by transition metals, such as those in the iron-sulfur minerals, were necessary, as is exhibited by the conserved organometallic enzymes of the most primitive metabolic pathways for carbon fixation and energy generation. This supports the iron-sulfur world hypothesis, wherein the formation of pyrite from pyrrhotite may have provided enough reducing power to catalyze these fundamental redox reactions [20,21]. However, the pyrrhotite-pyrite transformation coupled with redox reaction is not a sufficient condition for the building of the iron-sulfur world. As Schoonen *et al.* pointed out [25], the pyrrhotite-pyrite couple is not capable of reducing CO_2_, although it is termodinamically favorable, due to the energetically unfavorable electron transfer from the pyrrhotite valence band to the LUMO (lowest unoccupied molecular orbital) of CO_2_, especially at higher temperatures. In consequence, to evaluate the pyrrhotite system in the context of iron-sulfur world hypothesis, we studied its redox properties and design an experimental system that overcome the limitations of the FeS/FeS_2_ couple.

In our experiments, the initial goal was to mimic the oxidoreductase activity of the fermentative enzyme lactate dehydrogenase (LDH) and to connect the pyruvate-lactate redox reaction to the FeS/pyrite system (1). This reaction is essential for explaining the behavior of lactate-pyruvate in sulfur rich mediums.

Pyruvate + FeS + H_2_S → Lactate + FeS_2_ + H_2_ (1)

In a biochemical system, the LDH enzyme catalyzes the redox interconversion between pyruvate and lactate. In bacteria, there are two types of LDH: NADH-dependent and NADH-independent. In the first group, the pyruvate resulting from the glycolysis is reduced to lactate, regenerating the nicotinamide adenine dinucleotide (NAD^+^) from the reduced nicotinamide adenine dinucleotide (NADH) produced in the earlier step. In the second group, which mostly consists of anaerobic bacteria, the LDH does not need the cofactor NADH, and the lactate is used as a carbon source in the subsequent steps of metabolism [26]. One of these non-NADH dependent LDH is the lactate cyctochrome c reductase, which uses a Fe(III)-porphyrin complex as an electron acceptor to yield pyruvic acid and Fe(II)-porphyrin. 

**Figure 4:**
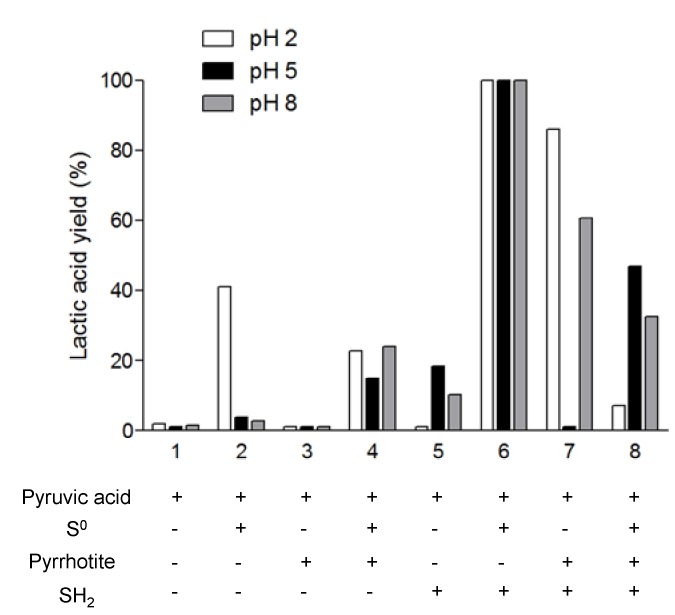
Lactic acid synthesis by pyruvic acid reduction coupled to iron sulfide/sulfur system. Role of pH and sulfur oxidation state.

To test the activity of the FeS/H_2_S system as a redox catalyst, we performed two different sets of experiments. First, we used pyruvic acid as a reagent to see whether this system can reduce it to lactic acid under different pH conditions. All of the reactions were carried out at 130 °C for 5 h in an anaerobic atmosphere. Figure 4 shows the percentage of lactic acid synthesis related to the initial amount of pyruvic acid present in the mixture.

If we look at the third column in Figure 4, we note that pyrrhotite *per se* is not able to promote the synthesis of lactic acid. However, when it is mixed with H_2_S at either an acidic or basic pH, there is a large reduction in pyruvic acid, greater than 50%. These results differ from those of Wang *et al.* [16], who found that the *in situ* precipitation of FeS reduced the pyruvic acid and that its mixture with H_2_S decreased the yield of the reaction.

**Figure 5:**
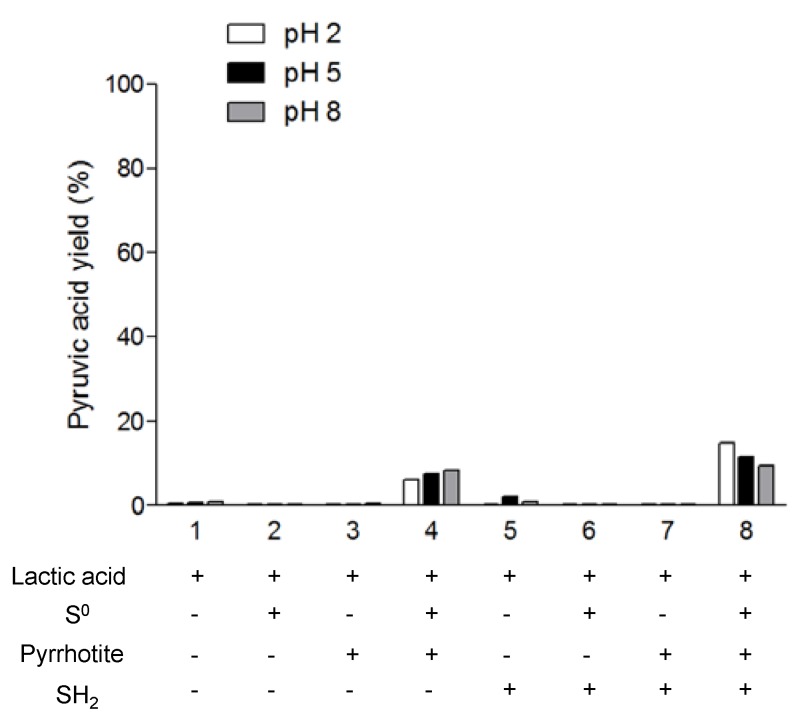
Pyruvic acid synthesis by lactic acid oxidation coupled with iron sulfide/sulfur system. Role of pH and sulfur oxidation state.

The highest rate of lactic acid production (100%) is obtained by mixing elemental sulfur (Sº) and H_2_S. If we compare these results with mixtures 2 and 4, where these reagents were used separately, we can see that the activity of the Sº and the H_2_S might be coupled, thereby enhancing the reaction. It is interesting to note that in mixture 2, Sº can reduce the pyruvic acid mainly at an acidic pH. Wang *et al.* [16] determined that Sº, at temperatures higher than 113 °C and at neutral pH, acts as an oxidant. Nevertheless, according to our results, Sº is not able to oxidize lactic acid (Figure 5), but it is a reducing agent at lower pH. 

We performed four reactions using pyrrhotite to clarify its role in the pyruvic acid reduction. The reactions correspond to conditions 3, 4, 7, and 8. Comparing them, we note that the highest yields of lactic synthesis are obtained in pyrrhotite/H_2_S. In this case, the amount of lactic synthesis is higher than in reaction 5, where only H_2_S was added. If the reaction medium contains pyrrhotite with Sº and H_2_S, the rate of pyruvic reduction is decreased compared with reaction 6. However, lactic acid synthesis in reaction 4, pyrrhotite + Sº, is enhanced at neutral and basic pH compared with reaction 2. Overall, we observe that in the presence of pyrrhotite at an acidic pH, the addition of Sº to the mixture decreases the rate of pyruvic acid reduction, though the rate is enhanced at neutral and basic pH. 

Taken together, our results demonstrate that the pyrrhotite is able to reduce pyruvic acid into lactic acid only in the presence of Sº and/or H_2_S. This result is important for explaining the reaction product in Section 3.2.

**Figure 6:**
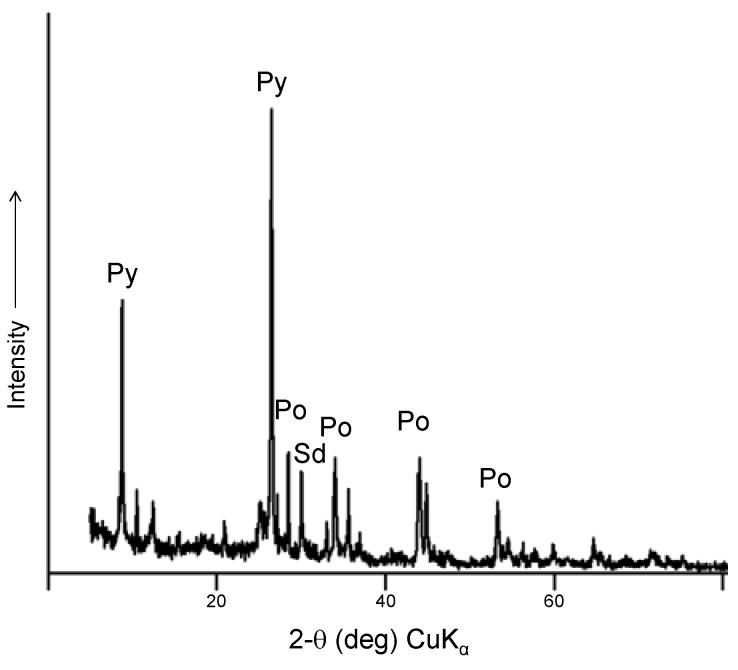
Powder X-ray diffraction of pyrrhotite ore after experiments. The identified phases include pyrite and siderite.

In the second set of experiments, we tested the reverse reaction, that is, the oxidation of lactic acid into pyruvic acid in sulfur rich systems. Figure 5 shows the percentage of pyruvic acid synthesis related to the initial amount of lactic acid. First, we note that the oxidation rates are lower than the reduction rates. In addition, the oxidation takes place only in the presence of pyrrhotite and Sº (reaction 4 and 8). There is no detectable pyruvic acid synthesis at reaction 7 (pyrrhotite + H_2_S); however, in the presence of elemental sulfur (reaction 8), the presence of H_2_S enhances the lactic acid oxidation compared with reaction 4 (pyrrhotite + Sº). In this case, our results are in agreement with those of Wang *et al.* [16] and support the idea of a coupled reaction system between FeS/Sº/H_2_S, mainly at acidic pH, and with a significant formation of pyrite, which is identified by XRD analysis of the solid material after the reaction (Figure 6). According to the results, the presence of H_2_S decreases the rate of oxidation at basic pH compared to acidic pH., The hypothesis used to explain this pH dependence is that the protonated state of the lactic acid affects the reaction. However, in our experiments, when we combined Sº with pyrrhotite, the effect of pH was the opposite of that in reaction 8; thus, it seems more reasonable to conclude that H_2_S is the factor causing the pH-dependent behavior of the reaction. In fact, at pH 2 and 5, the free sulfide is in the form of H_2_S, whereas at pH 8, it is in SH^−^ form. If we consider that there is a coupled reaction between FeS/Sº/H_2_S, the form of all the components under different pH conditions will affect the overall reaction. Additionally, the lactic acid could form stable complexes with iron, e.g., Fe(Lac)^+^ and Fe(Lac)_2_. The formation of iron-lactate complexes is an important factor in the explanation of the differences in the reactions. Lactic acid promotes iron mobilization from the mineral; simulations performed using Geochemist’s Workbench showed that the formation of Fe(III) lactate complexes is associated with pyrrhotite transformation in pyrite in the presence of Sº. This could explain the lack of oxidation to pyruvate. 

Considering the role of elemental sulfur in the oxidation of lactic acid, it is interesting to highlight that in the presence of pyrrhotite, it promotes the oxidation at low pH, which is in contrast with the results obtained for the reduction, where elemental sulfur decreases the yield of lactic synthesis. Thus, these results suggest that at 130 °C the Sº acts as an oxidant when the pH is acidic and as a reductant at neutral and basic pH. 

Overall, our results demonstrate that the pyrrhotite/Sº/H_2_S system has the ability to mimic the oxidoreductase activity and that it drives lactate-pyruvate redox chemistry in low temperature hydrothermal systems. The smaller yield of lactic synthesis in the presence of pyrrhotite, Sº and H_2_S in comparison with the same mixture without pyrrhotite (Figure 4) might be explained by the fact that in the presence of pyrrhotite the coupling reaction system is promoted at the same time as the reduction, and the oxidation is pH-dependent. 

### 3.2. Pyrrhotite/Iron Metal in Thioester Reductive Carboxylation: a Ferredoxin Mimic?

**Figure 7:**
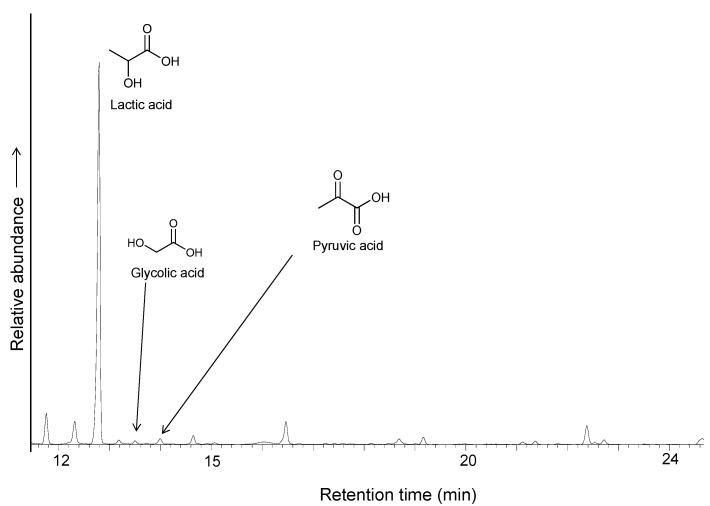
GC/MS chromatogram of the TMS derivatives of identified products obtained by carboxylation of ethylthioacetate coupled to the iron/pyrrhotite/sulfide electrochemical system.

Metallic iron is an extremely rare mineral in the modern Earth’s crust, mainly due to its instability in oxic conditions, but it may have been abundant in the early Earth before the rise of an oxygen-rich atmosphere. Iron is a good source of electrons, through its oxidation to Fe^2+^ (standard reduction potential, Eº = −440 mV). We constructed an electrochemical cell using a powder iron metal paste in water-HSO_4_^−^ at pH 5 as the anode, separated by a clay barrier from the cathode, which was formed by pyrrhotite containing 1 mmol of ethyl thioacetate and wetted with a NH_4_^+^ HCO_3_^−^ 50 mM solution (pH 9) containing 0.15 mmol of Na_2_S; we used the cell to test whether iron metal could be an additional source of reducing power and a supply of mobile iron under mild conditions. The system was connected to a power supply at 1.1 V under a nitrogen atmosphere in the circuit depicted in Figure 3; the current began at 17.4 µA, rose to a maximum of 23 µA and 1.87 V after 6 h and then decayed to a constant current of 10.5 µA. The organic solutes were analyzed after three days of standing in anoxic conditions at room temperature; the analysis shows a significant quantity of lactic acid (Figure 7) with an estimated yield of 6.5% of the added ethyl thioacetate. Pyruvic acid was also detected, as well as glycolic acid and glycine. Aliphatic amines and unidentified organic compounds, including sulfur-containing molecules, were formed.

No carbon chains greater than C_3_ were detected. The X-ray diffraction of pyrrhotite conducted after the experiment shows peaks of remaining pyrrhotite, pyrite, siderite and unidentified iron oxides, possibly wustite, ferrihydrite, and “green rust” (iron hydroxycarbonate). A control experiment performed without an external voltage source showed a significantly lower yield of lactic acid (less than 1%). An additional control experiment without thioester does not show formation of C_2_ or C_3_ organic compounds, suggesting that lactate/pyruvate are formed after carboxylation of thioacetate ester and that the formation of organic compounds directly from carbon dioxide is not possible under these conditions. 

The voltage source generates an electrochemical gradient and can supply additional electrons to the pyrrhotite-S^2−^/pyrite redox couple, which favors CO_2 _ reduction and is not an efficient electron acceptor due to its large overpotential (−2.22 V *vs.* SCE). The presence of iron-sulfur clusters can significantly reduce the CO_2_ reduction overpotential, promoting carbon fixation [20]. Under our chosen conditions, the anodic oxidation of iron metal could supply Fe^2+^ cations to the system, with subsequent formation of FeS_(aq)_, which constitutes the building blocks of crystalline forms of ferrous sulfides [27]. The soluble iron-sulfur complexes that constitute the aqueous form of FeS could have similar properties to the iron-sulfur clusters found in the metalloprotein ferredoxin, which may mediate the electron transfer reaction for the reductive carboxylation of thioacetic acid [28]. The formation of these protoferredoxins [8] could be favored by the formation of organic sulfur species, as ethanethiol, derived from ethyl thioacetate, which could form complexes with the formula [Fe_4_S_4_(SC_2_H_5_)_4_]^3−^. Under these conditions, the external source and the iron could constitute the electron donors necessary to keep the iron-sulfur complexes in a reduced state and to overcome the reduction potential of the pyruvate synthesis from CO_2_ and thioester (Eº = −500 mV). Hence, our experiment can be considered to be a biomimetic pathway to the biosynthesis of pyruvate from acetyl-CoA, promoted by PFOR and reduced ferredoxin [17]. The strongly favored presence of lactic acid, which is the major organic product of the experiment, can be explained by the formation of stable Fe-lactic acid complexes, which stabilize both the lactic acid and the iron in solution. 

**Figure 8:**
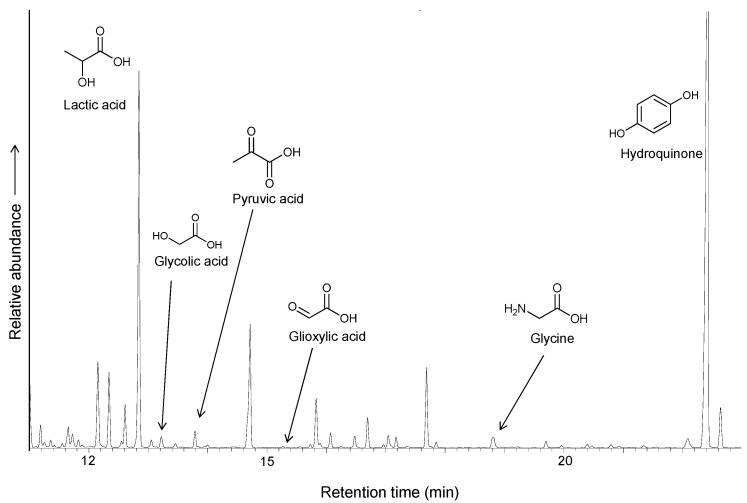
GC/MS chromatogram of the TMS derivatives of products obtained by carboxylation of ethylthioacetate coupled to the iron/pyrrhotite/sulfide electrochemical system, with hydroquinone as an additional electron donor.

The role of iron-sulfur clusters in the reductive carboxylation were demonstrated in the seminal work of Nakajima *et al.* [29], in which an alpha-keto acid is formed under mild conditions as an intermediate in the synthesis of phenylalanine from n-octyl-phenylthioacetate, catalyzed by a synthetic iron-sulfur cluster that models ferredoxin. Although the Nakajima reaction helped to conceptualize the possibility of iron-sulfur clusters as non-enzymatic catalysts and its potential role during life origin, the reaction is not geochemically extrapolable, as it has been performed in non-aqueous conditions and with hydrosulfite as electron donor (Eº = −0.660V). The electron donor role, as suggested in the Iron-Sulfur world theory, could be assumed by the pyrite formation from pyrrhotite in a geochemical environment [30]. In fact, the control experiment without an external voltage source shows a significantly lower yield in the formation of lactic acid, suggesting that a low potential electron donor could be necessary for the process. To test this possibility, we performed an experiment using the same electrochemical cell design but without using an external voltage source and adding 1 mmol of hydroquinone (Eº = −0.699V). Hydroquinone can act as analog of the biological ubiquinol and can perform electron transfer reactions on the surface of minerals [31]. The model biochemical reaction that motivates the selection of hydroquinone as an electron donor is the formation of pyruvate by direct carboxylation of acetic acid, promoted by (quinone) pyruvate dehydrogenase. The presence of hydroquinone promotes the synthesis of lactic acid, increasing the yield to 10.5% and suggesting that electrons can be transferred through iron sulfur clusters or surfaces, similar to the ubiquinol/iron-sulfur system in biochemistry (Figure 8). 

Our experiments show that iron metal is essential to the reaction, possibly as an electron source and soluble iron source. However, what is the role of pyrrhotite? A control experiment performed using silica sand instead of pyrrhotite does not show the formation of C_3_ derivatives, which indicates that pyrrhotite plays an essential role in the formation of pyruvate/lactate. Although the synthesis of pyruvate, promoted by FeS, has been reported by G.D. Cody *et al.* [32], the high CO pressure experimental conditions suggest a different mechanism. Under our mild conditions, two facts could help to explain the role of pyrrhotite. (i) The pyrrhotite structure is complex; it is a non-stoichiometric, iron defective sulfide, with surface vacancies and distortions [33]. The surface chemistry of pyrrhotite, although not widely studied, should be different than of troilite (its stoichiometric equivalent) and the synthetic FeS phases usually used in prebiotic chemistry. The electronic properties of pyrrhotite particles could allow them to be used as electron transfer conduits to the species in solution or to newly formed membraneous precipitates, in a electrochemical induced pathway similar to the photoelectrochemical formation of alpha-ketoglutaric acid from pyruvate, catalyzed by sphalerite particles [34]. (ii) The formation of pyrite in the presence of hydrogen sulfide, which, in synergy with the formation of soluble protoferredoxin clusters, could supply reducing power in the form of molecular hydrogen. This pyrite-pulled reaction connects the prebiotic chemistry with the biochemistry; this is an equivalent mechanism to that in which ferredoxin is maintained in a reduced state by hydrogen in chemolithotrophs [12]. Although the system shows considerable chemical complexity, and further work is necessary to explain the products obtained, the common key feature is the disequilibrium. We established an electrochemical reactor, with dissipation of a potential and pH gradient, together with the formation of new mineral precipitates, following the theory postulated by M. Russell [35,36]. The main difference is that the Russell theory proposed an exhalative submarine model that involves the mixture of two fluids at different temperature and pH: The Hadean ocean water and the hydrothermal fluid. The formation of membranous iron sulfide precipitate in the fluid interface promoted an environment where accumulation of clusters and organics and the dissipation of electrochemical gradients through membrane, promotes the protometabolic reactions. We propose in this experiment a fluid-rock interaction model in which iron metal and iron sulfur minerals promoted the formation of new species and the adequate environment for the carbon fixation, by formation of new iron solid species and soluble clusters. The presence of previously formed organic precursors (such as thioesters or alkyl thiols, in the reported experiment) is necessary to the increase of organic complexity in the mild conditions tested. 

**Figure 9:**
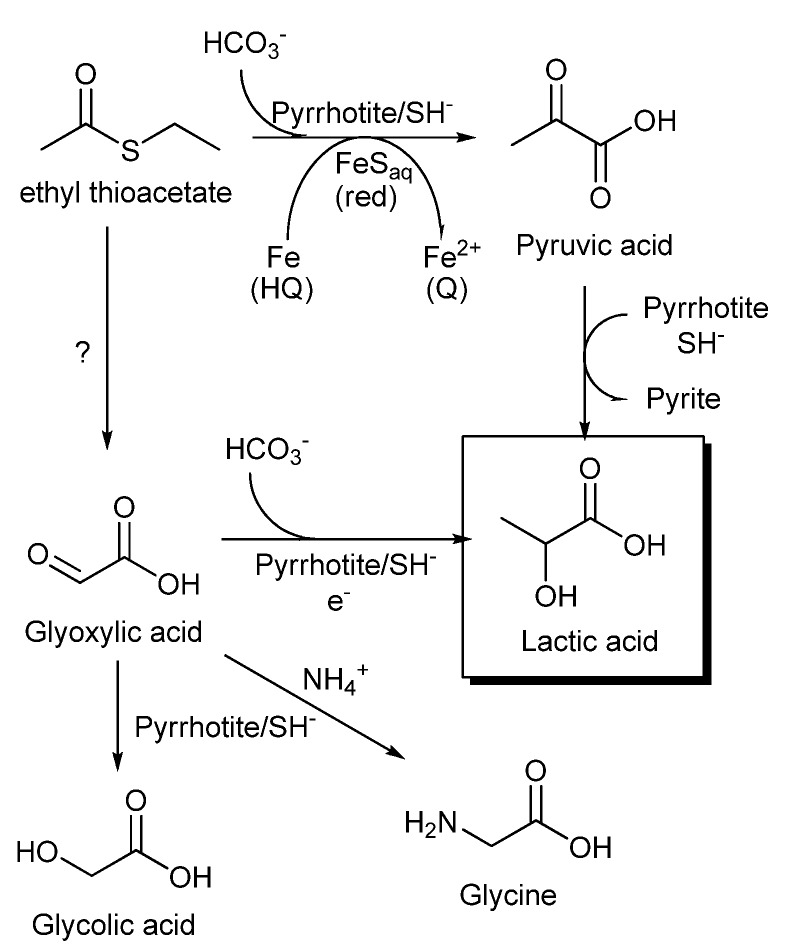
Proposed routes to the synthesis of lactic acid by carboxylation of an alkyl thioacetate ester. The synthesis of pyruvic acid could be regarded as a prebiotic analog of reductive carboxylation of acetyl CoA promoted by PFOR, with iron-sulfur clusters of ferredoxin as electron donors.

An interesting feature of the reaction is the formation of glycolic acid and glycine. The reductive amination of alpha-keto acids in the presence of ammonium to yield amino acids has been reported under mild conditions in the presence of Fe^2+^ [37]. In our results, we did not identify any amino acids larger than glycine. The glycine could be synthesized from glyoxylic acid by transamination using other amino acids as nitrogen donors [38] and by reductive amination in the presence of strong reducing agents [39]. The possible presence of glyoxylic acid as an intermediate under our conditions connects the pyrrhotite/electrochemical reduction with the synthesis of lactate, which is promoted by the highly reducing photo-generated conduction band electrons in the zinc sulfide mineral sphalerite [40]. This photo-electrochemical synthesis leads to the direct formation of lactate by a reaction between carbon dioxide and glyoxylic acid. It is possible that the electronic properties of pyrrhotite favor a similar pathway in the electrochemical cell (Figure 9), although oxalic acid was not identified and there was no evidence of thioacetate independent carbon fixation. It is interesting to note the lack of organic acids greater than C_3_, which suggests that there are no further reactions involving pyruvic acid or that reduction to lactic acid or degradation (for example, formation of oxaloacetate and degradation to pyruvate) are kinetically favored.

In summary (Figure 9), the formation of lactic acid under our experimental conditions can be explained through pyruvate synthesis by the reductive carboxylation of thioacetate ester, followed by a reduction to lactic acid (see Section 3.1). Additionally, the presence of glycolic acid and glycine could suggest alternative pathways, including the glyoxylate intermediary. Further experiments are necessary to explore the potential of electrochemical gradients in combination with sulfide minerals and soluble iron sulfide complexes in the origin of a biochemical system. The identification of unidentified amino- and thio- derivatives found in our experiments could help to explain the complex mechanism implicated in the biomimetic reductive carboxylation of a thioester. Aside from establishing the precise mechanism that this system uses for the formation of C_3_ acids from C_2_ precursors and carbon dioxide, our experiments show that iron sulfides in a non-equilibrium environment, characterized by the presence of electrochemical gradients and soluble and solid iron species in diverse oxidation states, could support biologically useful reactions. This supports the idea that transition metal sulfides play a role in the development of protometabolism. 

## 4. Conclusions

The natural iron sulfide pyrrhotite, in a soluble sulfur-rich environment and in the presence of newly formed mineral precipitates and soluble iron-sulfur clusters (whose formation was induced by iron metal anodic oxidation), can promote the reductive carboxylation of simple thioacetic acid esters to form pyruvate/lactate under mild conditions. The reaction could be regarded as an abiotic analog of the pyruvate synthesis promoted by pyruvate ferredoxin oxidoreductase and favored by the presence of low potential electron donors, such as hydroquinone, which suggests that the origin of ancient organic cofactors boosted the emergence of simple protometabolic systems. Additionally, the complexity of the resultant system does not exclude alternative pathways, such as reductive amination or alternative carbon fixation mechanisms. The results are consistent with the theories that place the iron sulfide species at the center of the geochemistry-biochemistry transition and show that the diversity of oxidation states of iron and sulfur and the morphologies of the solid iron phases, in a scenario that favors the electrochemical coupling by the proximity of diverse mineral forms, is fundamental for carbon fixation to the C_3_ compounds lactate/pyruvate from the C_2_ precursors (thioacetate or ethylthiol). This suggests that the dissipation of electrochemical gradients and the electronic properties of pyrrhotite and other iron sulfur species could promote the emergence of a thioester based biochemical system. 

## References

[1] Holm N.G. (1992). Marine Hydrothermal Systems and The Origin of Life.

[2] Harvey R.B. (1924). Enzymes of thermal algae. Science.

[3] Martin W., Baross J., Kelley D., Russell M.J. (2008). Hydrothermal vents and the origin of life. Nat. Rev. Microbiol..

[4] Cody G.D., Boctor N.Z., Brandes J.A., Filley T.R., Hazen R.M., Yoder H.S. (2004). Assaying the catalytic potential of transition metal sulfides for abiotic carbon fixation. Geochim. Cosmochim. Acta.

[5] Wächtershäuser G. (1988). Before enzymes and templates: theory of surface metabolism. Microbiol. Rev..

[6] Huber C., Wächtershäuser G. (1997). Activated acetic acid by carbon fixation on (Fe,Ni)S under primordial conditions. Science.

[7] Russell M.J., Hall A.J., Turner D. (1989). *In vitro* growth of iron sulphide chimneys: possible culture chambers for origin-of-life experiments. Terra Nova.

[8] Russell M.J. (1997). The emergence of life from iron monosulphide bubbles at a submarine hydrothermal redox and pH front. J. Geol. Soc. London.

[9] Martin W., Russell M.J. (2007). On the origin of biochemistry at an alkaline hydrothermal vent. Philos. T. Roy. Soc..

[10] Fuchs G. (2011). Alternative Pathways of Carbon Dioxide Fixation: Insights into the Early Evolution of Life?. Ann. Rev. Microbiol..

[11] Eck R.V., Dayhoff M.O. (1966). Evolution of the structure of ferredoxin based on living relics of primitive amino acid sequences. Science.

[12] Beinert H. (2000). Iron-sulfur proteins: ancient structures, still full of surprises. J. Boil. Inorg. Chem..

[13] Russell M.J., Martin W. (2004). The rocky roots of the acetyl-CoA pathway. Trends Biochem. Sci..

[14] Holm R.H., McCleverty J.A., Meyer T.J. (2003). Electron Transfer: Iron–Sulfur Clusters. Comprehensive Coordination Chemistry II.

[15] Russell M.J., Hall A.J., Kesler S.E., Ohmoto H. (2006). The onset and early evolution of life. Evolution of Early Earth’s Atmosphere, Hydrosphere and Biosphere-Constraints from Ore Deposits.

[16] Wang W., Yang B., Qu Y., Liu X., Su W. (2011). FeS/S/FeS2 redox system and its oxido reductase-like chemistry in the iron-sulfur world. Astrobiology.

[17] Furdui C., Ragsdale S.W. (2000). The role of pyruvate ferredoxin oxidoreductase in pyruvate synthesis during autotrophic growth by the Wood-Ljungdahl pathway. J. Biol. Chem..

[18] Ragsdale S.W., Pierce E. (2008). Acetogenesis and the Wood-Ljundahl pathway to CO_2_ fixation. Biochim. Biophys. Acta.

[19] De Duve C. (1991). Blueprint for a Cell: The Nature and Origin of Life.

[20] Cody G.D. (2004). Transition metal sulfides and the origins of metabolism. Ann. Rev. Earth Planet. Sci..

[21] Wächtershäuser G. (1990). Evolution of the first metabolic cycles. Proc. Natl. Acad. Sci. USA.

[22] For a detailed overview of the Gualba deposit. http://www.foro-minerales.com/forum/viewtopic.php?p=82307&highlight=gualba#82307.

[23] Hazen M.R., Deamer W.D. (2007). Hydrothermal Reactions of Pyruvic acid: Synthesis, Selection, and Self-Assembly of Amphiphilic Molecules. Origins Life Evol. B.

[24] Cooper G., Reed C., Nguyen D., Carter M., Wang Y. (2011). Detection and formation scenario of citric acid, pyruvic acid, and other possible metabolism precursors in carbonaceous meteorites. Porc. Natl. Acad. Sci. USA.

[25] Schoonen M.A.A., Xu Y., Bebie J. (1999). Energetics and kinetics of the prebiotic synthesis of simple organic acids and amino acids with the FeS-H_2_S/FeS_2_ redox couple as reductant. Origins Life Evol. B.

[26] Garvie E.I. (1980). Bacterial Lactate Dehydrogenases. Microbiol. Rev..

[27] Rickard D., Luther G.W. (2007). Chemistry of Iron Sulfides. Chem. Rev..

[28] Bonomi F., Werth M.T., Kurtz D.M. (1985). Assembly of FenSn(SR)2- (n = 2,4) in aqueous media from iron salts, thiols and sulfur, sulfide, thiosulfide plus rhodonase. Inorg. Chem..

[29] Nakajima T., Yabushita Y., Tabushi I. (1975). Amino acid synthesis through biogenetic-type CO_2_ fixation. Nature.

[30] Drobner E., Huber H., Wächtershäuser G., Rose D., Stetter K.O. (1990). Pyrite formation linked with hydrogen evolution under anaerobic conditions. Nature.

[31] Kung K., Mcbride M.B. (1988). Electron Transfer Processes Between hydroquinone and iron oxides. Clay. Clay Miner..

[32] Cody G.D., Boctor N.Z., Filley T.R., Hazen R.M., Scott J.H., Sharma A., Yoder H.S. (2000). Primordial car- bonylated iron-sulfur compounds and the synthesis of pyruvate. Science.

[33] Rosso K.M., Vaughan D.J. (2006). Sulfide mineral surfaces. Rev. Miner. Geochem..

[34] Guzman M.I., Martin S.T. (2009). Prebiotic metabolism: production by mineral photoelectrochemistry of alpha-ketocarboxylic acids in the reductive tricarboxylic acid cycle. Astrobiology.

[35] Russell M.J. (2007). The alkaline solution to the emergence of life: Energy, entropy and early evolution. Acta biotheor..

[36] Mielke R.E., Robinson K.J., White L.M., Mcglynn S.E., Mceachern K., Bhartia R., Kanik I., Russell M.J. (2011). Iron-Sulfide-Bearing Chimneys as Potential Catalytic Energy Traps at Life’s Emergence. Astrobiology.

[37] Huber C., Wächtershäuser G. (2003). Primordial reductive amination revisited. Tetrahedron Lett..

[38] Nakada H.I., Weinhouse S. (1953). Non-enzymatic transamination with glyoxylic acid and various amino acids. J. Biol. Chem..

[39] White R.H. (1983). A simple synthesis of (RS)-[2–2H] glycine by the reductive amination of glyoxylic acid. J. Labelled Compd. Rad..

[40] Guzman M.I., Martin S.T. (2010). Photo-production of lactate from glyoxylate: how minerals can facilitate energy storage in a prebiotic world. Chem. Commun..

